# The role of glucocorticoid, interleukin-1β, and antioxidants in prenatal stress effects on embryonic microglia

**DOI:** 10.1186/s12974-018-1079-7

**Published:** 2018-02-16

**Authors:** Jada Bittle, Hanna E. Stevens

**Affiliations:** 1 Department of Psychiatry, University of Iowa Carver College of Medicine, 1330 PBDB, 169 Newton Rd., Iowa City, IA 52246 USA; 2 Interdisciplinary Graduate Program in Neuroscience, University of Iowa, 356 Medical Research Center, Iowa City, IA 52242 USA; 3 Iowa Neuroscience Institute, University of Iowa Carver College of Medicine, 2312 PBDB, 169 Newton Rd., Iowa City, IA 52246 USA

**Keywords:** Prenatal stress, Embryonic brain, Microglia, Corticosterone, Interleukin-1β, Antioxidant

## Abstract

Maternal stress during pregnancy is associated with an increased risk of psychopathology in offspring. Resident immune cells of the brain, microglia, may be mediators of prenatal stress and altered neurodevelopment. Here, we demonstrate that neither the exogenous pro-inflammatory cytokine, interleukin-1β (IL-1β), nor the glucocorticoid hormone, corticosterone, recapitulated the full effects of prenatal stress on the morphology of microglial cells in the cortical plate of embryonic mice; IL-1β effects showed greater similarity to prenatal stress effects on microglia. Unexpectedly, oil vehicle alone, which has antioxidant properties, moderated the effects of prenatal stress on microglia. Microglia changes with prenatal stress were also sensitive to the antioxidant, N-acetylcysteine, suggesting redox dysregulation as a mechanism of prenatal stress.

## Introduction

Stress experienced by a mother during pregnancy is a risk factor for neuropsychiatric disorders including autism and schizophrenia in offspring [1–5]. Maternal immune activation (MIA) during pregnancy is also a risk factor for neuropsychiatric disorders [6–8], suggesting that common maternal and offspring neurodevelopmental mechanisms may be involved. The mechanisms by which lasting, neurobiological alterations are induced in the developing fetal brain by maternal physiological changes have yet to be elucidated and are critically important for translational efforts to prevent psychiatric disorders.

Models of MIA have highlighted the importance of alterations in immune factors, such as microglia, in fetal neurodevelopment. Microglia contribute to neurodevelopment and may be a significant component of effects of MIA and prenatal stress [9]. In the adult mouse brain, microglia act similar to macrophages, traveling to the site of injuries and cleaning up cellular debris; however, their function during brain development is less clear. Microglial cell progenitors enter the central nervous system after the first fetal week and play a vital role in cortical development by regulating the size of the neural progenitor cell pool [10]. Prenatal stress and MIA both change microglia in postnatal rodent brain [11, 12]. At postnatal day 1, prenatal stress increased ramified microglia but reduced amoeboid microglia in cortical regions. In fetal brain, prenatal stress and interleukin-6 induced the most significant changes in the density of multivacuolated microglia [13]. MIA caused increased numbers of microglia in the adult brain, alongside changes in their activation [6]. The maternal physiology underlying these microglial changes is not understood.

One maternal physiological component of prenatal stress is elevation of plasma corticosterone levels [11]. Previous work implicates corticosterone as a key mediator in effects of prenatal stress on the embryo [14] and has been shown to influence microglia [9]. Prenatal stress also elevates pro-inflammatory cytokines, including interleukin-1β (IL-1β) [15], and the embryonic brain is known to be sensitive to maternal cytokines [13, 16]. MIA upregulates multiple pro-inflammatory cytokines in the fetal brain, including IL-1β, IL-6, IL-17, IL-13, MCP-1, and MIP α, hours after MIA, but only IL-1β was shown to be elevated in the fetal brain 24 h following the induction of MIA [8, 17]. IL-1β plays a key role in regulation of microglia during their development [8]. Glucocorticoids and IL-1β are important candidate mediators for prenatal stress effects on the embryonic brain.

Here, we sought to elucidate maternal factors that underlie the effects of prenatal stress on embryonic microglia in a mouse model. To assess physiological candidates of a commonly used prenatal stress model that showed the greatest effect on microglia at embryonic day 14 (E14) [13], we injected the stress hormone, corticosterone, and the pro-inflammatory cytokine, IL-1β, beginning on E12. We characterized microglia found within the developing cortical plate by morphology at E14, following 2 days of exposures [13, 18]. The timing of this exposure coincides with the first wave of invasion of the microglia into the neocotex [19]. By E14, microglial cells are present throughout the brain parenchyma and are known to play a crucial role in the development of the cortex, including regulating the size of the neural progenitor cell pool [10]. While not predicting that each factor could recapitulate stress effects in a reductionist way, we assessed whether they would have similarity to the effects of prenatal stress on microglia.

## Methods

### Mice

GAD67-GFP+ knock-in mice were bred on a CD1 background and housed in accordance with the University of Iowa Institutional Animal Care and Use Committee (IACUC) policies. Following the detection of a vaginal plug on embryonic day 0 (E0), pregnancies were monitored and dams were singly housed from E12 onward.

### Treatment

On E12 and E13, three times daily, pregnant dams were injected intraperitoneally (ip) with 100 ng/200 μl interleukin-1β (R&D Systems) in saline, 200 μl 0.9% saline, 500 μg/200 μl corticosterone (Sigma) in sunflower seed oil (Sigma), or 200 μl sunflower seed oil. Dosing was based on previous restraint stress studies with IL-1β and corticosterone (i.e., [20]).

### Prenatal stress

Three groups were exposed to prenatal stress: PS, PS oil, and PS NAC (Table 1). To induce stress, pregnant dams were placed in a Plexiglass restraint apparatus and under bright lights for 45 min, three times a day, starting on E12 [13, 21, 22] at approximately three and a half hour intervals. Experiments were performed simultaneously across groups that were compared.

**Table 1:** Experimental conditions and number of litters and fetuses used in each

	Litters	Fetuses
Non-stress		
NS	4	7
Corticosterone	3	6
Oil	4	5
Saline	4	7
IL-1β	3	6
NS NAC	3	6
Prenatal stress		
PS	4	6
PS oil	3	6
PS NAC	4	6

N-acetylcysteine (NAC) is a common antioxidant supplement that contributes to replenishing the endogenous antioxidant, glutathione. NAC has been used to demonstrate the role of redox dysregulation in disease models, typically administered chronically [23]. In order to provide for sufficient antioxidant during the periods of interest (E12–E14) without the potential stress of a dietary change during the period of interest, pregnant dams were provided water ad libitum with either regular water or NAC-infused water (PharmaNAC; 2 mg/ml of water) for the duration of pregnancy (from E0 onward).

### Immunohistochemistry

Pregnant dams were euthanized and E14 brain tissue collected and post-fixed in 4% paraformaldehyde/phosphate buffered saline. Following transfer to 20% sucrose, tissue was embedded and cryo-sectioned (Leica, CM1900, Bannockburn, Illinois) at 25 μm. Tissue sections were incubated in 10% goat serum/PBS++ blocking solution (with 0.025% Trition X-100, 0.0125% Tween 20) at room temperature for at least 1 h. They were then incubated overnight with 5% goat serum/PBS++ primary antibody, anti-Iba-1 (WAKO; rabbit polyclonal 1:300–600) followed by Alexa dye-conjugated secondary antibody (1:500–1000; Molecular Probes) in 5% goat serum/PBS++ incubation for 2 h. Tissue section slides were then cover slipped using DAPI mounting medium (4′,6-diamidino-2-phenylindole, Vector Laboratories, #H-1200) and observed under an epifluorescent microscope (Zeiss).

### Microglia assessment

**Fig. 1:**
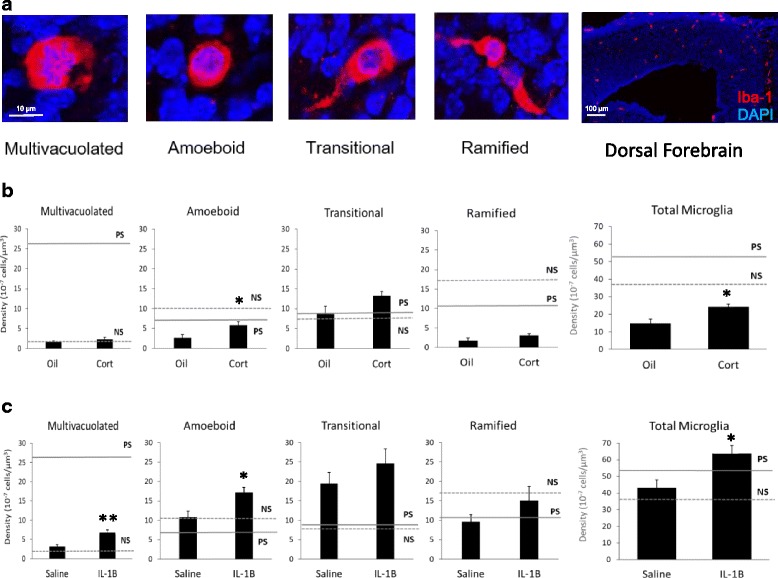
Effects of stress factors on total microglia and morphological subtype density in E14 cortical plate. Potential maternal stress factors included glucocorticoid stress hormone, corticosterone (Cort), and pro-inflammatory cytokine, IL-1β. **a** Microglia morphological subtypes (Iba1 in red and DAPI in blue). Scale bars 10 and 100 μm. **b** Corticosterone increased total and amoeboid microglia, but did not recapitulate the most significant effect of prenatal stress (solid line), increased multivacuolated microglia. **c** IL-1β increased total and amoeboid microglia. IL-1β increased multivacuolated microglia, but not to the extent of prenatal stress. (*p < 0.05 and **p < 0.01 compared to oil or saline). Previous findings on microglia density indicated by dotted line for non-stress (NS) and solid line for prenatal stress (PS) levels, [13].

**Fig. 2:**
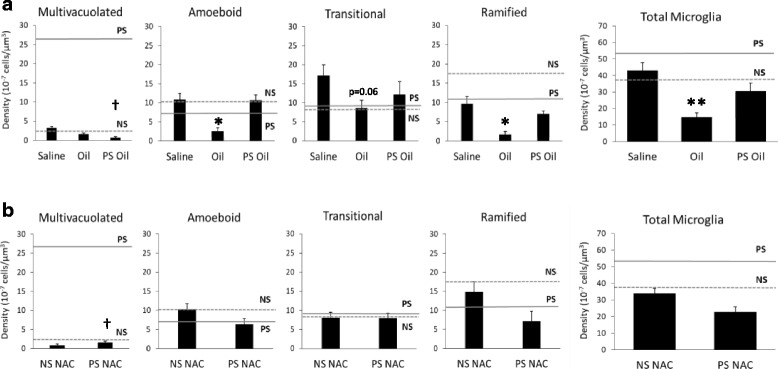
Sunflower seed oil and N-acetylcysteine (NAC) moderated the effects of prenatal stress on multivacuolated microglia. **a** Oil reduced total microglia density and the density of amoeboid and ramified microglia compared to saline. Oil with prenatal stress (PS oil) reduced prenatal stress-induced levels (solid line) of multivacuolated microglia. **b** Concomitant NAC with prenatal stress (PS/NAC) resulted in no difference in microglia density for any subtype compared to NS/NAC, but reduced prenatal stress-induced levels (solid line) of multivacuolated microglia. (*p < 0.05 and **p < 0.01 compared to saline or NS/NAC). Previous findings on microglia density indicated by dotted line for non-stress (NS) and solid line for prenatal stress (PS) levels, [13].(^†^*p* < 0.05 compared to PS)

Stereological cell counting was performed using the optical fractionator approach and unbiased counting rules with 3-dimensional 200 × 150 × 10 μm counting frames, on a 500 × 300 μm grid using a 40× objective lens (Stereoinvestigator; MBF Biosciences). Stereological counting to determine cell density, displayed as means and standard errors of the mean (Figs. 1 and 2), was performed in 3–5 serial coronal sections (every 20th section) of the embryonic neocortical primordium (cortical plate) as previously described [13]. Density was calculated by dividing the number of each type or all microglia by cortical plate (E14) volume, as computed by the Cavalieri volume estimation approach (mean volume 2.5 ± 0.17 mm^3^) and was highly correlated with total number of microglia. Density was the measure of choice to allow comparison to previously published data [13].

Microglial morphology was categorized as before [13] (Fig 1a), with amoeboid cells identified by a round soma and normal nucleus, transitional cells with a single process, ramified cells with two of more processes, and multivacuolated cells with multiple vacuoles and/or pyknotic nuclei.

### Statistical analysis

All statistics were performed using SPSS v.24 (IBM, IL, USA). A one-way analysis of variance (ANOVA) was performed to identify differences across groups for each morphology. One ANOVA was performed across the corticosterone, IL-1β, oil, and saline groups. To correct for multiple comparisons, post hoc Tukey and Tamhane’s T2 (for unequal variances) tests were used to reveal significant differences in the density of total microglia and each subtype between the stress factor (corticosterone and IL-1β) and vehicle groups (oil and saline). A second ANOVA was performed across the oil, PS oil, and NAC oil groups, and appropriate post hoc tests were performed. Embryo sex was not assessed—we previously identified no effect of sex on embryonic microglia morphology [13].

## Results

### Corticosterone increased total and amoeboid microglia density

Repetitive corticosterone injection of pregnant dams resulted in increased overall density of microglia by 64.9% (*p* < 0.05) and increased density of amoeboid microglial cells by 114.7% (*p* < 0.05), compared to vehicle control (Fig. 1b). While we previously demonstrated that prenatal stress substantially increased multivacuolated microglia [13], corticosterone did not recapitulate this effect (Fig. 1b, solid line).

### IL-1β increased total, amoeboid, and multivacuolated microglia density

Repetitive IL-1β injection in pregnant dams increased the overall density of microglial cells by 47.4% compared to vehicle control (*p* < 0.05, Fig. 1c). IL-1β also increased the density of amoeboid microglia by 59.2% (*p* < 0.05) and of multivacuolated microglia by 109.9% (*p* < 0.01, Fig. 1c). Although IL-1β significantly increased the density of multivacuolated microglia compared to saline, IL-1β did not recapitulate the full effect of PS on multivacuolated microglia (Fig. 1c, solid line).

### Oil vehicle moderated the effects of prenatal stress on microglia

Interestingly, differences in the two vehicle controls—sunflower seed oil and saline—prompted a secondary analysis. Sunflower seed oil, a commonly used vehicle, reduced the overall density of microglia and the density of amoeboid and ramified microglia compared to saline (Fig. 2a). Amoeboid microglia were reduced by 303.0% (*p* < 0.05), ramified microglia by 444.9% (*p* < 0.05), and total density of microglia by 192.7% (*p* < 0.01), after oil injection compared to saline (Fig. 2a). Furthermore, when sunflower seed oil was administered prior to each episode of prenatal stress (PS oil), multivacuolated microglia density was reduced substantially compared to prenatal stress alone (*p* < 0.05, solid line, Fig. 2a).

### Antioxidant moderated the effects of prenatal stress on microglia

These unexpected results led to additional assessment of prenatal stress mechanisms. We hypothesized that antioxidant enhancement, one documented mechanism of sunflower seed oil [24, 25], may reduce effects on embryonic microglial. We tested this hypothesis by administering a different supplement with antioxidant properties, N-acetylcysteine (NAC), to pregnant dams. Concomitant NAC with prenatal stress (PS/NAC) had no effect on microglial density for any of the subtypes compared to NAC alone (NS/NAC, Fig. 2b). Similar to sunflower seed oil, there was a substantial reduction (*p* < 0.05) in the prenatal stress-induced increase of multivacuolated microglia with NAC administration.

## Discussion

Model system studies offer strong evidence of a causal role for prenatal stress in disrupting critical neurodevelopmental processes in offspring that have impacts over the life span [1, 26]. The effects of prenatal stress on microglial may represent a component of the developmental pathophysiology of stress-related neurobiological changes in the embryonic brain. We found here that corticosterone and IL-1β each had distinct influences on embryonic microglial cells compared to prenatal stress. Aside from maternal diffusible factors, our data also revealed that prenatal stress influences on microglia may involve downstream cellular mechanisms influenced by these mediators such as redox regulation. We found that two manipulations with overlapping antioxidant properties, sunflower seed oil and the antioxidant, N-acetylcysteine (NAC), moderated microglial changes with prenatal stress in the same way. These results are consistent with research that demonstrates that maternal oxidative stress status directly influences fetal microglia activation through gene expression changes and that maternal antioxidant status, in the case of maternal pretreatment with NAC, has protective effects on fetal microglia [27]. These findings suggest that multiple physiological changes, including the dysregulation of endocrine, immune/inflammatory, and redox pathways, likely underlie prenatal stress as a risk factor for neurodevelopmental disruptions, including microglia [9, 18].

The effects of each individual stress factor used here were similar to each other, both increasing the total density of microglia which broad prenatal stress did not [13]. These manipulations coincided with the entry of microglia into the embryonic brain [19], leaving open the possibility that increased microglia entry/relocation can be affected by maternal physiological changes. Similar to prenatal stress, IL-1β also increased multivacuolated microglia density, albeit to a much smaller extent (Fig. 1c), with changes in the phagocytic action of microglia and its commensurate role in neurodevelopment [28] as a potential consequence. In conjunction with the total density increase and no decrease in other morphologies with IL-1 β, these changes did not resemble shifts of microglia from one form to another. Our lab has shown previously that the pro-inflammatory cytokine, IL-6, is sufficient and necessary for a prenatal stress-like shift to more multivacuolated morphologies. IL-1β, therefore, may intersect with prenatal stress effects rather than play a primary mediating role.

Corticosterone, similarly, increased total embryonic microglia similar to previous studies [9] and increased amoeboid microglia density. Glucocorticoids have a complex relationship with immune function, often showing not only suppression of immunity and inflammation but also, in more limited circumstances, activation of these pathways [29]. Our results suggest that in comparison to its vehicle alone, corticosterone enhanced some aspects of the immune component of the embryonic brain. Our findings of corticosterone influences on microglia, however, must be interpreted in light of the effects of oil vehicle alone, which may have prevented changes that corticosterone may otherwise have induced. This oil, like other vegetable oils, has antioxidant and anti-inflammatory properties [24, 25]. In fact, we found that the co-administration of this oil together with prenatal stress was sufficient to prevent microglial changes. Anti-inflammatory aspects of this oil may also be involved in its impacts on microglia disruption, a process we have highlighted here with IL-1β and previously with IL-6 [13]. Anti-oxidant and anti-inflammatory mechanisms are highly overlapping, with redox processes responding to inflammation and vice versa. We found converging evidence for redox mechanisms, with no alteration of microglial density when prenatal stress was co-administered with N-acetylecysteine. Intriguingly, our results demonstrating alterations of embryonic microglia with IL-1β also support redox mechanisms, based on the importance of reactive oxygen species for IL-1β’s activation of microglia [30, 31]. These convergent findings support the importance of redox dysregulation in the effects of prenatal stress [32, 33].

The results of this study indicate that isolated physiological stress mediators, corticosterone, and IL-1β, induced increases in embryonic microglia, but not shifts in morphology as previously seen after prenatal stress. The buffering of redox processes negated embryonic microglia changes, implicating this among multiple stress-dependent mechanisms. Exploring maternal stress physiology can deepen our understanding of immune, endocrine, and redox pathways of importance during development and elucidate risks for neuropsychiatric disorders. Understanding these mechanisms and pathways will provide the basis for developing preventive approaches, similar to folic acid supplementation during pregnancy, that may safeguard normal development.

## Abbreviations

Cort: Corticosterone
E12, E13, E14: Embryonic day 12, 13, 14
IL-1β: Interleukin-1 beta
MIA: Maternal immune activation
NAC: N-acetylcysteine
NS: Non-stressed
PS: Prenatally stressed
